# Editorial: Cardio-respiratory-brain integrative physiology: interactions, mechanisms, and methods for assessment

**DOI:** 10.3389/fncom.2025.1664088

**Published:** 2025-08-12

**Authors:** Tijana Bojić, Luca Faes, Steffen Schulz, Tomislav Stankovski

**Affiliations:** ^1^Laboratory for Radiation Chemistry and Physics, Institute for Nuclear Sciences Vinča-National Institute of Serbia, University of Belgrade, Belgrade, Serbia; ^2^Department of Engineering, University of Palermo, Palermo, Italy; ^3^Faculty of Technical Sciences, University of Novi Sad, Novi Sad, Serbia; ^4^Charité Competence Center for Traditional and Integrative Medicine (CCCTIM), Charité–Universitatsmedizin Berlin, Corporate Member of Freie Universität Berlin and Humboldt-Universität zu Berlin, Berlin, Germany; ^5^Faculty of Medicine, Ss Cyril and Methodious University-Skopje, Skopje, North Macedonia; ^6^Department of Physics, Lancaster University, Lancaster, United Kingdom

**Keywords:** brain, heart, lungs, interactions, physiology, mechanisms, networks, methods

**Figure 1:**
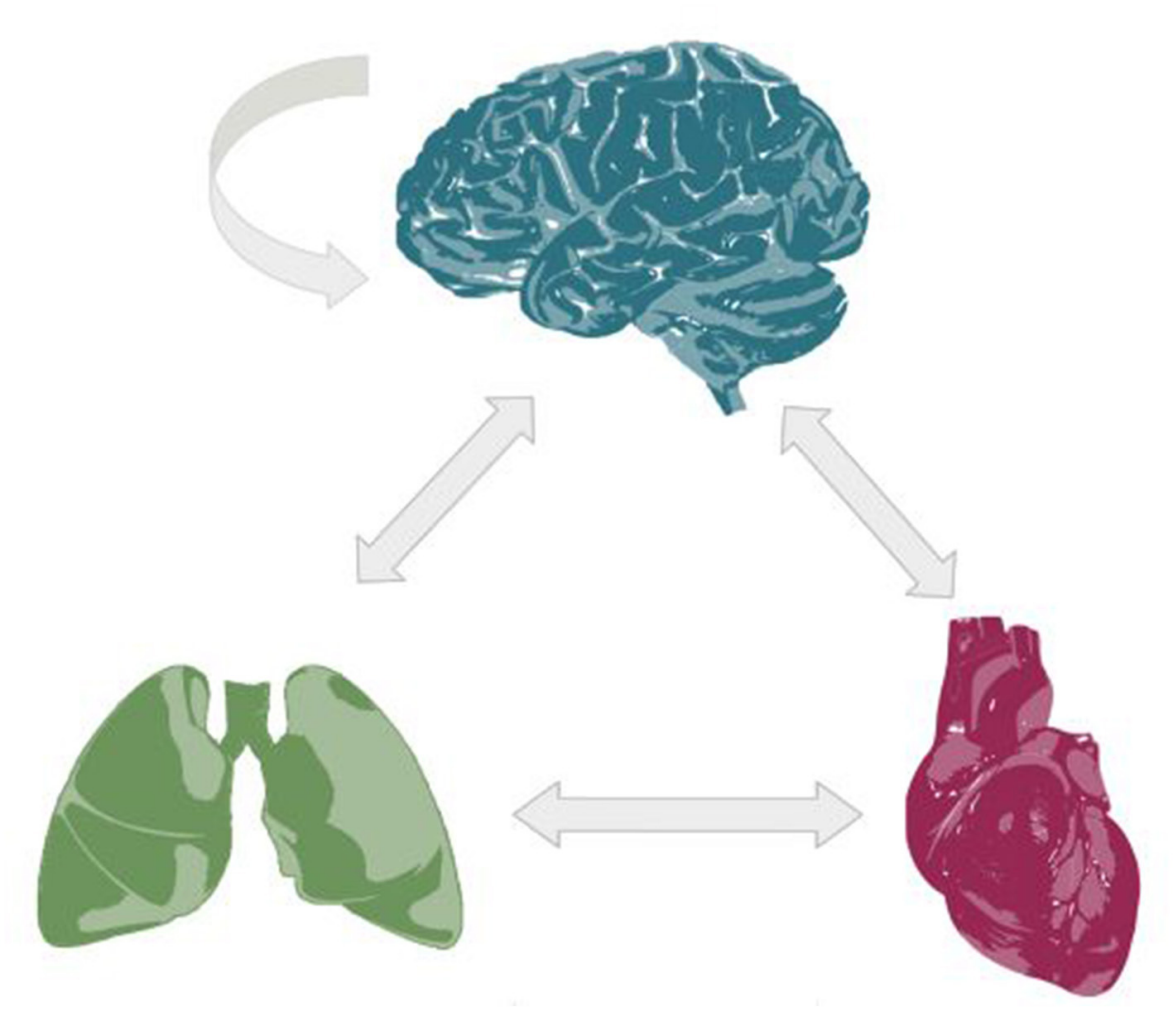
Physiological network for the brain-heart-lungs interactions.

The discipline of cardio-respiratory-brain integrative physiology investigates the complex interactions among the heart, lungs, and brain, emphasizing how these systems work together to sustain overall stability and balance within the body. The cardiorespiratory coupling was studied in some recent studies (Schulz et al., 2013; Acampa et al., 2021; Kapidžić et al., 2014), including the respiratory sinus arrhythmia (Hirsch and Bishop, 1981), cardiorespiratory phase synchronization (Schäfer et al., 1998) and coupling functions (Iatsenko, Bernjak; Lukarski et al., 2020). These coupling forms are primarily regulated by the brain, which is the core node of the well-known functional brain-heart interplay (Candia-Rivera et al., 2025) and mediates interactions with the lungs through the respiratory centers (Faes et al., 2015; Stankovski et al., 2016) (Figure 1). Interactions among the cardiac, respiratory, and brain systems are essential for understanding the central-autonomic network, which regulates the balance between parasympathetic and sympathetic nervous activity to adapt to different physiological and environmental challenges. Although progress has been made, gaps still exist in fully understanding the exact mechanisms and pathways driving these interactions, especially in disease states where imbalances can result in negative health effects. Recent research efforts are increasingly employing sophisticated signal processing and neuroimaging methods to explore these complex dynamics, but a complete understanding of the integrated physiology of these systems is still evolving.

The Research Topic aim was to enhance the understanding of cardio-respiratory-brain interactions by encouraging the development and implementation of innovative methodological approaches (Voss et al., 2008; Stankovski et al., 2012; Stankovski, 2013; Faes et al., 2015). The objective was to investigate both the physiological and pathological dimensions of these interactions from the perspective of biophysics, bioengineering, artificial intelligence, signal processing and clinical medicine. At the same time, the Research Topic covers theoretical concepts of the interaction networks and the underlying mechanisms (Pikovsky et al., 2001; Bashan et al., 2012; Stankovski et al., 2017). By addressing these areas, the Research Topic uncovered fresh perspectives on cardiovascular function, autonomic nervous system dynamics, and brain-heart interactions, paving the way for enhanced diagnostics and treatments.

This Research Topic emphasized the value of multidisciplinary approaches in integrative physiology. By fostering collaboration across the scientific community, it deepened understanding of fundamental science questions and advanced practical solutions for clinical diagnostics and therapies. These diverse perspectives provided a comprehensive picture of integrative physiology.

The Research Topic includes eleven manuscripts, in very diverse formats, ranging from original research, methods, case reports, clinical trials, and review articles. The majority of which, seven out of eleven, are original research. Schizas et al. present a research about the self-organized temporal criticality and multifractal complexity in order to describe the brain-heart-lungs complex systems and their interactions in the form of optimal empirical transfer of information. In doing so, they present both theoretical and methodological aspects, as they also applied this framework for time series analysis. Schulz et al. introduce a directionality index derived from high-resolution joint symbolic dynamics for evaluation of information transfer in multivariate physiological networks. To validate this index, various non-linear and linear, bivariate and multivariate coupled systems based on Gaussian autoregressive models were employed. The key benefits of these new directionality indices include their robustness to non-stationary time series, ability to effectively detect couplings, scale invariance, and independence from time series length, model order, and significance level procedures. In their research manuscript Orphanides et al. propose spectral-based ECG derived respiration method, which is non-invasive and low-cost, and can extract the breathing rate from the heart rate. They applied their method on data for studying whole-night sleep and the different sleep stages. Kalauzi et al. introduce a method to detect respiratory frequency rhythm using human alpha phase shifts. They implemented their innovative methodological framework on EEG oscillations from a cohort of young, healthy participants in both wakeful and drowsy states. Reyes-Lagos et al. used the information from short-term recurrence quantification analysis of pulse-respiration quotient to investigated the effect of diabetes on the cardiorespiratory dynamics. In their research manuscript Tischer et al. studied personalized auricular vagus nerve stimulation and discuss how the beat-to-beat deceleration is oredominant in systole-gated stimulation on inspiration. Their pilot study demonstrated that auricular vagus nerve stimulation can influence heart rate, thereby modulating parasympathetic activity. Karlen-Amarante et al. investigate how the postinspiratory and preBötzinger complexes affects the respiratory-sympathetic coupling in mice after and before chronic intermittent hypoxia. Abdollahpur et al. investigate the transient and steady-state effects of parasympathetic and sympathetic stimulation on f-wave characteristics in atrial fibrillation patients for a tilt test. Their findings indicate that alterations in f-wave frequency with head-up and head-down tilt may be associated with changes in sympathetic activity, with parasympathetic activity potentially modulating these effects rather than independently driving changes in fibrillatory rate. Karemaker describes a compelling case study of a non-athlete female with pronounced respiratory sinus arrhythmia. The report noted heart rate fluctuations from over 100 to under 60 bpm, often beat-to-beat, with a repetitive pattern tied to respiration, indicating an extreme manifestation of respiratory sinus arrhythmia. By conducting a randomized controlled trial Ladriñán-Maestro et al. investigated the effects of an inspiratory muscle fatigue protocol on respiratory muscle strength and heart rate variability in healthy young individuals. Their findings suggest that acute inspiratory muscle fatigue adversely affects heart rate variability and inspiratory muscle strength in this population. Alvarez-Araos et al. reviewed the baroreflex and chemoreflex link in high-altitude exposure, with a particular focus on how this can play a role on the exercise performance.

Insights from the eleven manuscripts in this Research Topic span broad overview of the new findings on the study of the Cardio-Respiratory-Brain network. They will stimulate further discussion and investigations on this and other integrative physiological networks.
